# Cryo-EM Structures of Group II Intron Ribonucleoprotein Complexes

**DOI:** 10.1063/4.0001013

**Published:** 2025-10-27

**Authors:** Sarah A Starcovic, Evan R Cramer, Aaron R Robart

**Affiliations:** West Virginia University, Morgantown, WV, USA

## Abstract

Group II introns are ribozymes that catalyze self-splicing reactions through two competing pathways: a non- physiological hydrolytic reaction and a branched splicing reaction that generates intron lariats. These introns often encode an intron-encoded protein (IEP) that facilitates splicing. A primitive subtype, known as IIC introns, relies on the IEP for efficient intron lariat formation. Following splicing, the intron associates with the IEP to form a ribonucleoprotein (RNP), which is essential for reverse splicing and site-specific integration into a DNA substrate. This high target specificity suggests potential applications in gene-editing technologies. Due to their RNP-dependent nature, IIC introns provide a unique model for understanding the evolutionary transition from self-splicing ribozymes to the RNP-dependent splicing complexes found in eukaryotic cells. Here, we present cryo-electron microscopy images of RNP formation and partial integration of the RNP into a DNA substrate. Previous studies revealed that the wild-type intron RNA forms dimers through structural contacts, complicating RNP assembly and data processing. To address this, we introduced mutations that disrupt dimerization, improving sample preparation and streamlining structural analysis. Interestingly, disrupting these dimer contacts also enhances the catalytic activity of the intron RNA. These structural insights support our broader objective of understanding splicing pathway selection. By leveraging these findings, we aim to optimize this system, contributing to the expanding repertoire of gene-editing technologies.

